# Supplementary material for: Top predators constrain mesopredator distributions
Source: Nat Commun. 2017 May 23;8:15469. doi: 10.1038/ncomms15469 (PMC5457510; doi:10.1038/ncomms15469)
Supplement: Supplementary Information — Supplementary Figures, Supplementary Tables, Supplementary Methods and Supplementary References [file ncomms15469-s1.pdf]

Supplementary Table 1. Piecewise regression results for the relationship between mesopredator bounty data and distance to the edge (85% kernel) of the top predator's range. Estimates for the intercept (Intercept) and slope of first line segment (Line 1 85%) are shown with standard errors (SE). Numbers for line 2 85% represent the difference in slopes between the first and second line segment within each regression (see Fig. 3). Line 2 is significant (*P*<0.05) when the slope of line 2 differs from the slope of line 1 at the breakpoint.

		Estimate	SE	*t*-value	*P*
North America grey wolf (top predator)					
	Intercept	-0.40180	0.00237	-169.81	<0.001
	Line 1 85%	0.00009	0.00001	6.58	<0.001
	Line 2 85%	-0.00011	0.00002	-4.95	<0.001
North America coyote (mesopredator)					
	Intercept	0.96225	0.11345	8.482	<0.001
	Line 1 85%	-0.00412	0.00050	-8.265	<0.001
	Line 2 85%	0.00405	0.00124	3.255	<0.01
Europe grey wolf (top predator)					
	Intercept	-0.39620	0.00336	-117.918	<0.001
	Line 1 85%	0.00005	0.00002	2.302	<0.05
	Line 2 85%	-0.00024	0.00005	-4.574	<0.001
Europe golden jackal (mesopredator)					
	Intercept	0.60426	0.08237	7.336	<0.001
	Line 1 85%	-0.00585	0.00058	-10.162	<0.001
	Line 2 85%	0.00602	0.00135	4.466	<0.001
Australia East dingo (top predator)					
	Intercept	0.00378	0.13479	0.028	0.98
	Line 1 85%	0.00762	0.00075	10.128	<0.001
	Line 2 85%	-0.01587	0.00210	-7.56	<0.001
Australia East red fox (mesopredator)					
	Intercept	1.13405	0.12137	9.344	<0.001
	Line 1 85%	-0.00452	0.00067	-6.787	<0.001
	Line 2 85%	0.00254	0.00193	1.317	0.19
Australia West dingo (top predator)					
	Intercept	0.06411	0.23171	0.277	0.78
	Line 1 85%	0.01106	0.00154	7.189	<0.001
	Line 2 85%	-0.00778	0.00367	-2.121	<0.05
Australia West red fox (mesopredator)					
	Intercept	1.00485	0.10100	9.949	<0.001
	Line 1 85%	-0.00479	0.00056	-8.617	<0.001
	Line 2 85%	0.00413	0.00192	2.148	<0.05

Supplementary Table 2. Break point summary. Position refers to the distance of the break point from the edge of the top predators range (see Fig. 3).

	Position	2.5% CI (km)	97.5% CI (km)
North America grey wolf (top predator)	241.13	211.84	300.38
North America coyote (mesopredator)	383.56	311.64	450.13
Europe grey wolf (top predator)	208.25	48.29	244.91
Europe golden jackal (mesopredator)	214.09	183.19	241.99
Australia East dingo (top predator)	301.54	237.08	397.34
Australia East red fox (mesopredator)	307.84	56.75	416.51
Australia West dingo (top predator)	258.64	-33.99	416.80
Australia West red fox (mesopredator)	319.77	-15.03	371.87

Supplementary Table 3: Additional notes on data sources.

**Data sources**	**Data types**	**Relevant provinces (year) and details of hunting units.**	**Other relevant notes**
Government of Saskatchewan	Coyote and grey wolf fur returns from wild harvests.	Saskatchewan (1982-2011 across 136 hunting units)	• Detailed fur return data collected at the individual wildlife management zone level by the Government of Saskatchewan each year based on payments made to trappers and hunters.<br>• The coyote data was previously used and validated[1].
Hunting associations	Golden jackal and grey wolf fur returns from wild harvests.	Bulgaria (2004-2009 across 255 hunting units)	• Detailed fur return data collected at the individual wildlife management zone level by hunting associations each year.<br>• The grey wolf and golden jackal data was previously used and validated[2].
Hunting associations	Golden jackal and grey wolf fur returns from wild harvests.	Serbia (2000-2008 across 148 hunting units)	• Detailed fur return data collected at the individual wildlife management zone level by hunting associations each year.<br>• The grey wolf and golden jackal data was previously used and validated[2].
Government of Queensland	Dingo and red fox fur returns from wild harvests.	Southern two thirds of Queensland (1950-1951 based on published maps)	• Detailed fur return data providing the location of where dingoes and red foxes were killed across the southern two thirds of Queensland. Each location represented five dingoes or five red foxes were killed.<br>• The dingo and red fox data was previously used and validated[3].

Supplementary Methods: *R* Code used to assess relationships between top predators and mesopredators in North America, Europe and Australia. In North America the top predator is the grey wolf (*Canis lupus*) and the mesopredator is the coyote (*Canis latrans*). In Europe the top predator is the grey wolf (*Canis lupus*) and the mesopredator is the golden jackal (*Canis aureus*). In Australia (East and West) the top predator is the dingo (*Canis dingo*) and the mesopredator is the red fox (*Vulpes vulpes*).

```
#################################################################################

# Newsome et al. (2017). Nature Communications.

# Script to look at meso- and top-predator piecewise regression, with both datasets scaled (z-scored)

#################################################################################


# Load package

library(SiZer)


# Load data


Pred85.scale = read.csv("pred_edge_data_scaledData.csv", header=T)


# functions for piecewise regression that subsets data (per country)


PW.meso <- function(dd,country){

  data.country <- subset(dd, Country==country)

  out<- piecewise.linear(data.country$Distance, data.country$Meso.scale, middle = 1, CI = TRUE, sig.level = 0.05)

  return(out)

        }

PW.top <- function(dd,country){

  data.country <- subset(dd, Country==country)
```

```
  out<-  piecewise.linear(data.country$Distance,  data.country$Top.scale,  middle  =  1,  CI  =
TRUE,  sig.level  = 0.05)

  return(out)

}


################################################################################

# Australia

# mesopredator

# East


AustEastPW85.meso  <- PW.meso(Pred85.scale,  "AustEast")

AustEastPW85.meso

sum.AustEast85.meso<-summary(AustEastPW85.meso$model);  sum.AustEast85.meso



# West 85

AustWestPW85.meso  <- PW.meso(Pred85.scale,  "AustWest")

AustWestPW85.meso

sum.AustWest85.meso<-summary(AustWestPW85.meso$model);  sum.AustWest85.meso



#top predator

# East


AustEastPW85.top  <- PW.top(Pred85.scale,  "AustEast")

AustEastPW85.top

sum.AustEastPW85.top<-summary(AustEastPW85.top$model);sum.AustEastPW85.top
```

```
# West

AustWestPW85.top  <- PW.top(Pred85.scale,  "AustWest")

AustWestPW85.top

sum.AustWestPW85.top<-summary(AustWestPW85.top$model);  sum.AustWestPW85.top



################################################################################

# Nth America

#mesopredator

AmerPW85.meso  <- PW.meso(Pred85.scale,  "NthAmerica")

AmerPW85.meso

sum.AmerPW85.meso<-summary(AmerPW85.meso$model);sum.AmerPW85.meso



#top predator

AmerPW85.top  <- PW.top(Pred85.scale,  "NthAmerica")

AmerPW85.top

sum.AmerPW85.top<-summary(AmerPW85.top$model);  sum.AmerPW85.top



################################################################################
```

Supplementary Figure 1. Bubble plot of residuals from the North America grey wolf (top predator) regression model (Supplementary Table 1) plotted against their spatial co-ordinates. Black dots are negative residuals, and grey dots are positive residuals; all scaled to size based on residual values.

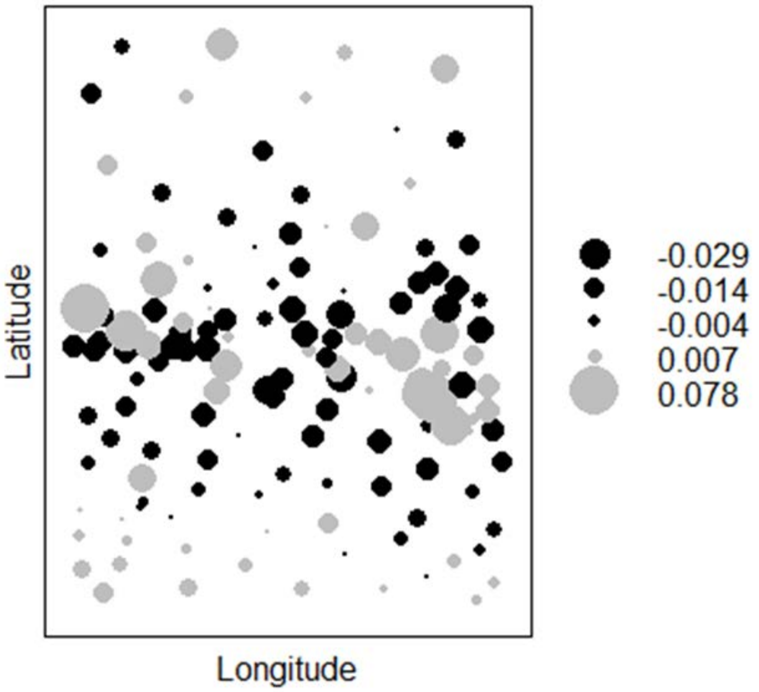

Supplementary Figure 2. Bubble plot of residuals from the North America coyote (mesopredator) regression model (Supplementary Table 1) plotted against their spatial co-ordinates. Black dots are negative residuals, and grey dots are positive residuals; all scaled to size based on residual values.

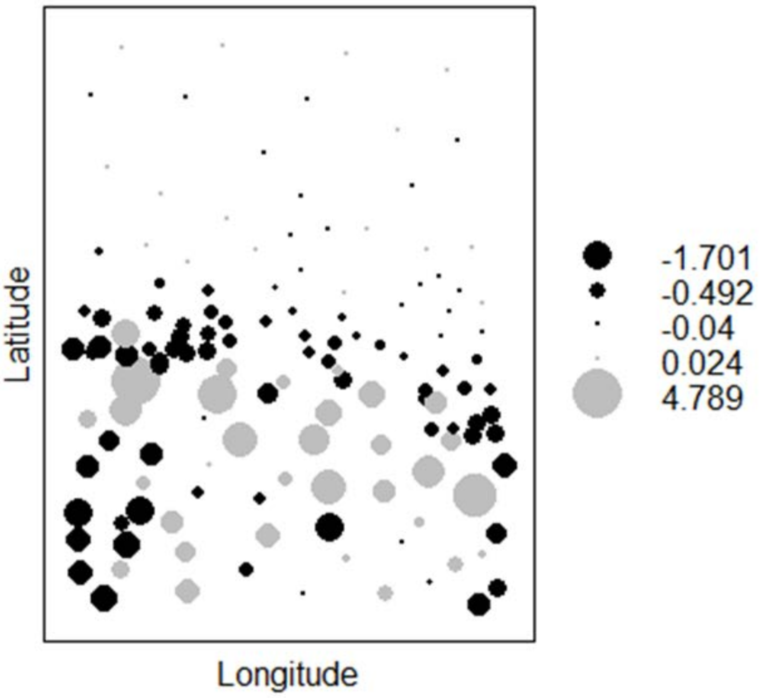

Supplementary Figure 3. Bubble plot of residuals from the Europe grey wolf (top predator) regression model (Supplementary Table 1) plotted against their spatial co-ordinates. Black dots are negative residuals, and grey dots are positive residuals; all scaled to size based on residual values.

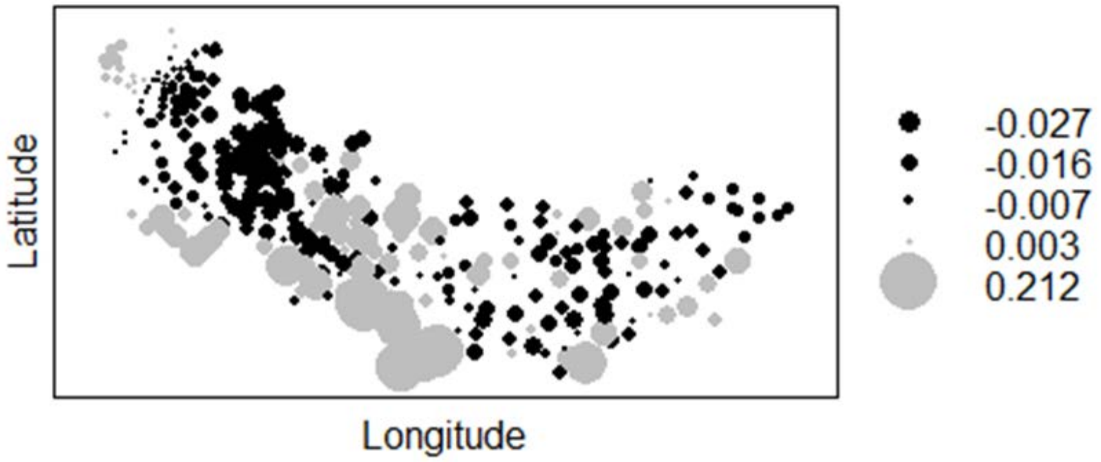

Supplementary Figure 4. Bubble plot of residuals from the Europe golden jackal (mesopredator) regression model (Supplementary Table 1) plotted against their spatial co-ordinates. Black dots are negative residuals, and grey dots are positive residuals; all scaled to size based on residual values.

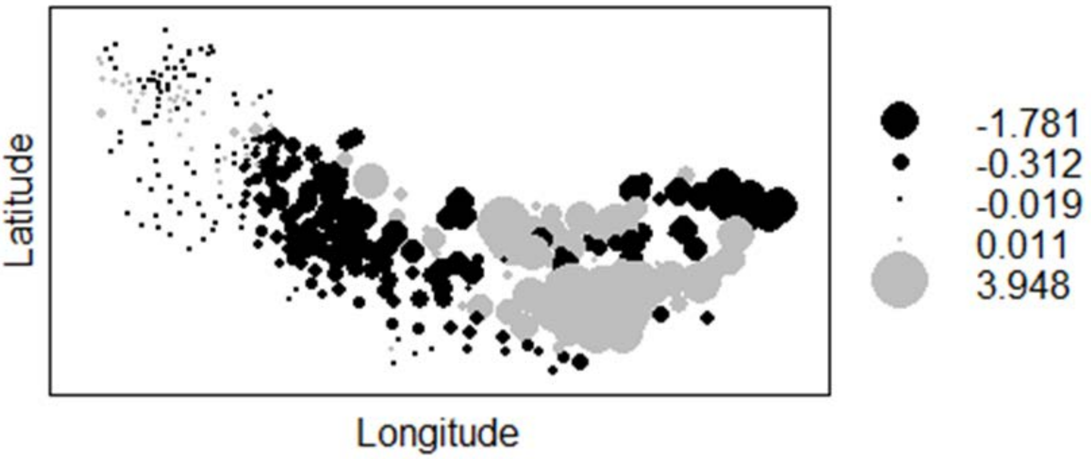

Supplementary Figure 5. Bubble plot of residuals from the Australia East dingo (top predator) regression model (Supplementary Table 1) plotted against their spatial co-ordinates. Black dots are negative residuals, and grey dots are positive residuals; all scaled to size based on residual values.

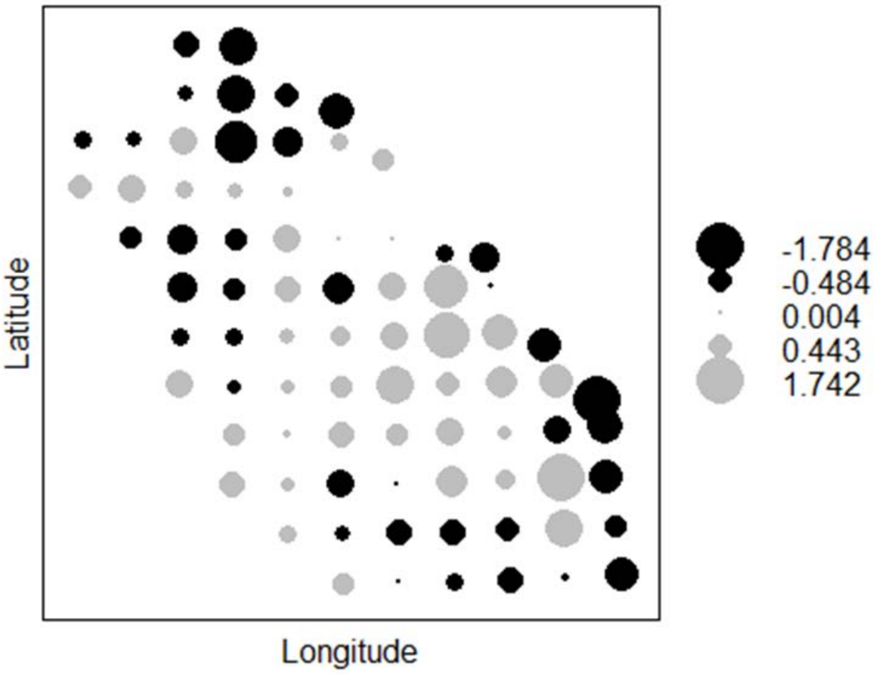

Supplementary Figure 6. Bubble plot of residuals from the Australia East red fox (mesopredator) regression model (Supplementary Table 1) plotted against their spatial co-ordinates. Black dots are negative residuals, and grey dots are positive residuals; all scaled to size based on residual values.

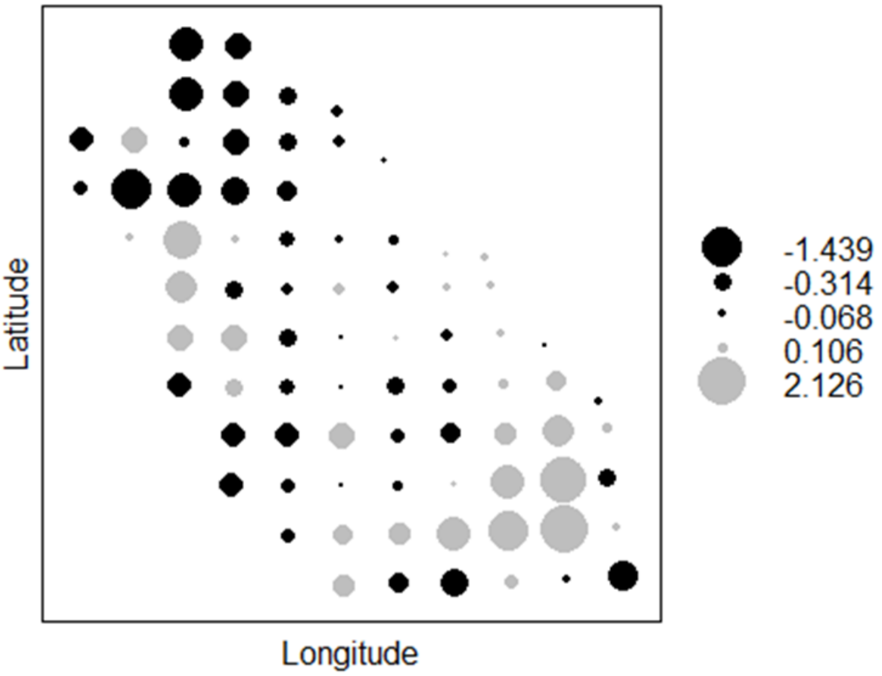

Supplementary Figure 7. Bubble plot of residuals from the Australia West dingo (top predator) regression model (Supplementary Table 1) plotted against their spatial co-ordinates. Black dots are negative residuals, and grey dots are positive residuals; all scaled to size based on residual values.

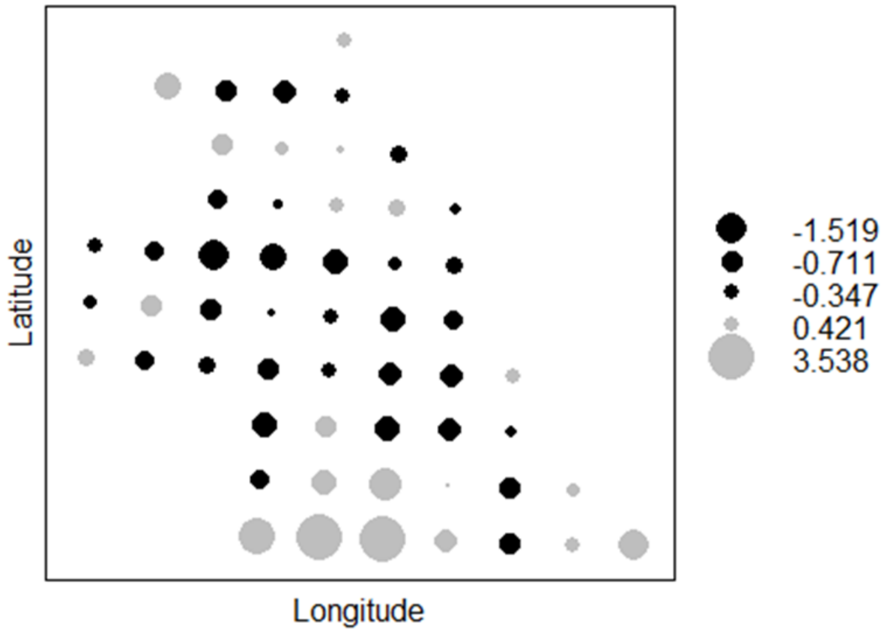

Supplementary Figure 8. Bubble plot of residuals from the Australia West red fox (mesopredator) regression model (Supplementary Table 1) plotted against their spatial co-ordinates. Black dots are negative residuals, and grey dots are positive residuals; all scaled to size based on residual values.

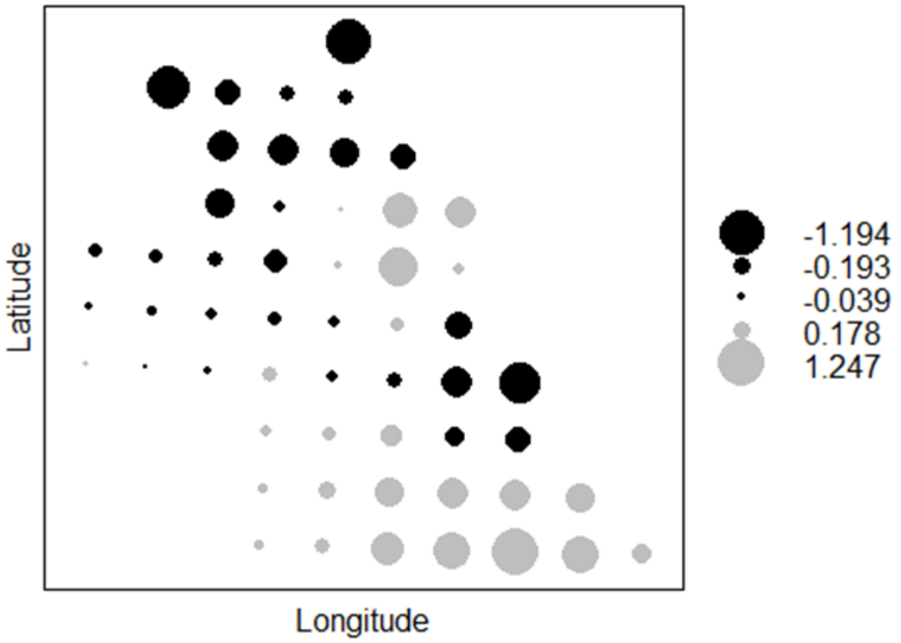

Supplementary References

1. Newsome, T. M. & Ripple, W. J. A continental scale trophic cascade from wolves through coyotes to foxes. *J. Anim. Ecol.* **84,** 49–59 (2015).
2. Krofel, M., Giannatos, G., Cirovic, D. & Newsome, T. M. Golden jackal expansion in Europe: a case of mesopredator release triggered by continent-wide wolf persecution? *Hystrix Ital. J. Mammal.* doi:10.4404/hystrix-28.1-11.
3. Letnic, M. *et al.* Does a top predator suppress the abundance of an invasive mesopredator at a continental scale? *Glob. Ecol. Biogeogr.* **20,** 343–353 (2011).
